# Impaired artery elasticity predicts cardiovascular morbidity and mortality- A longitudinal study in the Vara-Skövde Cohort

**DOI:** 10.1038/s41371-023-00867-1

**Published:** 2023-10-04

**Authors:** Gábor Szaló, Margareta I. Hellgren, Matthew Allison, Ying Li, Lennart Råstam, Karin Rådholm, Entela Bollano, Daniel A. Duprez, David R. Jacobs, Ulf Lindblad, Bledar Daka

**Affiliations:** 1 Primary Health Care, School of Public Health and Community Medicine, Institute of Medicine, Sahlgrenska Academy, University of Gothenburg, Gothenburg, Sweden; 2 The Skaraborg Institute, Skövde, Sweden; 3 Division of Preventive Medicine, Department of Family Medicine, University of California San Diego, La Jolla, CA USA; 4 Biostatistics, School of Public Health and Community Medicine, Institute of Medicine, Sahlgrenska Academy, University of Gothenburg, Gothenburg, Sweden; 5 Department of Clinical Sciences Malmö, Lund University, Lund, Sweden; 6 Department of Health, Medicine and Caring Sciences, Faculty of Medicine and Health Sciences, Linköping University, Linköping, Sweden; 7 Department of Cardiology, Sahlgrenska University Hospital, Gothenburg, Sweden; 8 Cardiovascular Division, Department of Medicine, University of Minnesota, Minneapolis, MN USA; 9 Division of Epidemiology and Community Health, School of Public Health, University of Minnesota, Minneapolis, MN USA

**Keywords:** Preventive medicine, Myocardial infarction, Risk factors

## Abstract

It is still debated whether arterial elasticity provides prognostic information for cardiovascular risk beyond blood pressure measurements in a healthy population. To investigate the association between arterial elasticity obtained by radial artery pulse wave analysis and risk for cardiovascular diseases (CVD) in men and women. In 2002–2005, 2362 individuals (men=1186, 50.2%) not taking antihypertensive medication were included. C2 (small artery elasticity**)** was measured using the HDI/Pulse Wave CR2000. Data on acute myocardial infarction or stroke, fatal or non-fatal, was obtained between 2002–2019. Cox- regression was used to investigate associations between C2 and future CVD, adjusting for confounding factors such as age, sex, systolic blood pressure, heart rate, HOMA-IR (Homeostatic Model Assessment for Insulin Resistance), LDL- cholesterol, CRP (C-Reactive Protein), alcohol consumption, smoking and physical activity. At baseline, the mean age of 46 ± 10.6 years and over the follow-up period, we observed 108 events 70 events in men [event rate: 5.9%], 38 in women [event rate: 3.2%]. In the fully adjusted model, and for each quartile decrease in C2, there was a significant increase in the risk for incident CVD by 36%. (HR = 1.36, 95% CI: 1.01–1.82, *p* = 0.041). The results were accentuated for all men (HR = 1.74, 95% CI: 1.21–2.50, *p* = 0.003) and women over the age of 50 years (HR = 1.70, 95% CI: 0.69–4.20). We showed a strong and independent association between C2 and CVD in men. In women after menopause, similar tendencies and effect sizes were observed.

## Background

Aging is the most important factor in the deterioration of arterial elasticity and increased arterial stiffness [1–3]. Other important factors that influence arterial elasticity are smoking, alcohol consumption, inflammation, atherosclerosis and chronic diseases such as diabetes mellitus, impaired glucose metabolism, dyslipidemia [2, 4, 5].

Atherosclerotic plaque formation is an essential mechanism to the development of cardiovascular disease (CVD) and begins with a dysfunctional endothelium [6]. Before, and parallel with, atherosclerotic plaque formation, degeneration of elastin material and an increase in collagenous material, mainly in tunica media, cause increased aortic impedance and early wave reflections in the vascular tree. Then, aortic stiffening raises systolic blood pressure, pulse pressure, and the left ventricular (LV) load and decreases coronary perfusion, leading to myocardial ischemia [4, 7].

Identifying individuals with early vascular changes can help stratify CVD risk and is pivotal to public health strategies. High blood pressure is a major risk factor for CVD [8]. However, there is also a paradigm shift in the early detection of cardiovascular disease from blood pressure to investigating pulsatile flow and measuring arterial elasticity [9]. In fact, longitudinal studies have shown that measures of arterial elasticity provide important prognostic information regarding CVD risk beyond arterial blood pressure measurement [10–14]. A few studies have examined the predictive value of aortic pulse wave velocity (PWV) for future CVD in a general population. Duprez et al. investigated associations between pulse wave analysis and emerging CVD [12].

We investigated whether arterial elasticity, measured by pulse wave analysis, predicts incident CVD in a Swedish study population free of overt disease at baseline.

## Methods

### Population

**Fig. 1:**
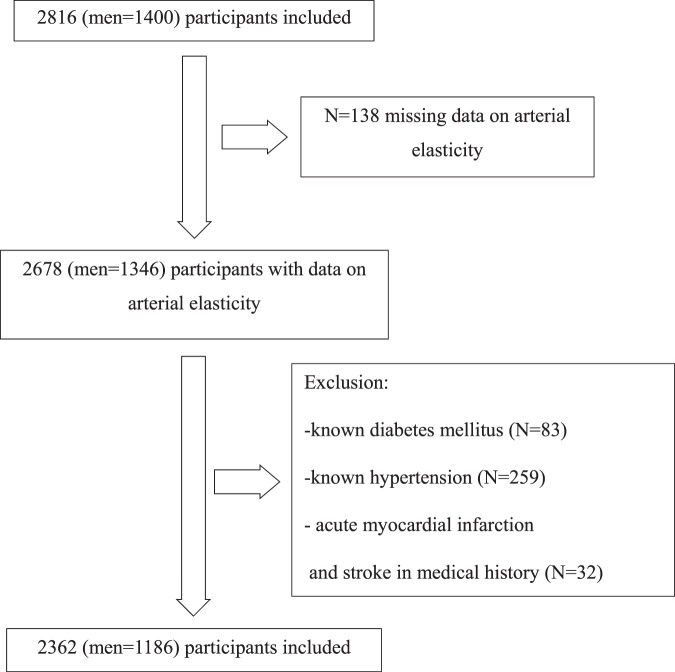
Study population.

This longitudinal observational study is based on participants from the Vara-Skövde cohort in the Skaraborg project [15]. To identify early signs of CVD and risk factors for the development of CVD, a total of 2816 individuals (men=1400) were randomly selected based on the census and were examined between 2002 and 2005. Arterial elasticity indexes were measured in 2678 individuals. Participants with prevalent acute myocardial infarction (AMI) and stroke (*N* = 32) at baseline were excluded from the analyses. Hypertensive medication can interfere with the elasticity indices significantly; thus, we chose to exclude participants with known hypertension (*N* = 259) or diabetes mellitus (DM) (*N* = 83) (Fig. 1). In total, we included 2362 individuals in our study (men=1186). All participants were followed from the baseline examination until the first cardiovascular event or death caused by CVD or were censored or end of the observation on December 31st, 2018.

### Measurements

Body height, weight, pulse and blood pressure were measured following standard procedures [15, 16], and hypertension was defined as systolic blood pressure (SBP) ≥ 140 or diastolic blood pressure (DBP) ≥ 90 [17]. Blood pressure was measured in a lying position after five minutes of resting. Two measurements were performed one minute apart and the mean value was used in the study. The measurement of blood pressure was made at the same time point as the pulse wave record.

Information on the medical history and lifestyle was collected using validated questionnaires. The level of physical activity was self-reported and estimated using the Leisure Time Physical Activity (LTPA) instrument, which has been validated in several previous studies [18, 19].

Fasting plasma insulin was analyzed using an enzyme-linked immunosorbent assay (ELISA). Insulin resistance was defined using the Homeostatic Model Assessment of Insulin Resistance (HOMA- IR) [20]. Diagnosis for diabetes mellitus was based on the 1999 WHO recommendation [21]. Assays for LDL cholesterol, triglyceride, C- reactive protein (CRP) and creatinine were performed using standard methods. Arterial elasticity was measured based on pulse contour analysis of the radial artery waveform using the HDI/Pulse Wave^TM^ CR-2000 (Eagan, Minnesota) and C2 (small artery elasticity) were estimated [22, 23]. Higher values of C2 indicate more compliant arteries. Quartiles of C2 were defined based on the distribution of C2 for men and women separately. Quartile 1 for C2 is defined as the lowest, quartile 4 for C2 is defined as the highest value for arterial elasticity.

### Outcome

Cardiovascular disease was defined as acute myocardial infarction (ICD-10: I21, I22, I23, I25) and/or stroke (ICD-10: I60, I61, I63, I64) (fatal and/or non-fatal). The data on CV morbidity were obtained from the Hospital Discharge Registers. The information for CV mortality and all-cause mortality was obtained from the Swedish Cause of Death Register. These registers are a valid alternative to revised hospital discharge and death certificates. We define CV events as a summary of CV mortality and morbidity. We use CV mortality and all-cause mortality in separate analyses.

### Statistics

Statistical analyses were performed using IBM SPSS 26. All our statistical analyses were two-sided. The *p* value for the statistically significant test result was *p* ≤ 0.05. Mean and standard deviation (SD) were presented for continuous variables. For categorical values, we used median and quartiles. According to our preliminary analyses, C2 had a skew- distribution with significant differences between men and women. Thus, we chose to define C2 quartiles separately for women and men. Kaplan-Meier curves and Cox- regression were used to investigate the association between quartiles of C2 and CV events. We examined the association between quartiles of C2 and CV mortality and all-cause mortality with Kaplan-Meier curves and Cox- regression in separate analyses. Cox regression analysis with adjustments for possible confounders were performed in different models. Model 1 was adjusted by age, sex, systolic blood pressure as a continuous variable and heart rate. Model 2 included adjustments for all the variables in model 1, adding HOMA-IR, LDL-cholesterol as a continuous variable and CRP. In model 3, model 2 was included and lifestyle factors such as alcohol consumption, smoking and physical activity were added to this model. In a sensitivity analysis, we used adjustments for pulse pressure instead of systolic blood pressure. We added statin medication for the model 2 in an alternative adjustment.

To investigate the possible modification of menopause, we performed a stratified analysis for women (<50 vs. ≥50 years old), with a parallel breakdown in men, to assess consistency with the overall findings.

We performed C-statistics for model 3 to analyze the incremental role of C2 compared with common CV risk factors. Model 3 includes age, sex, heart rate, SBP, HOMA- IR, LDL cholesterol, CRP, smoking, alcohol consumption and physical activity.

## Results

The average age of participants in this study at baseline was 46 ± 10.6 years. The average follow-up time was 14.8 $\pm$ 2.4 years. The population’s characteristics showed significant differences between women and men in most cardiovascular and metabolic variables (Table 1). The values and distribution of C2 differed by sex with C2 quartiles for men, median 8.87 ml × mmHg^−1^ × 100 (IQR: 6.53–11.01) and C2 quartiles for women, median 6.83 ml × mmHg^−1^ × 100 (IQR: 4.57–8.99). C2 indices were significantly higher for men and highest in both women and men under 50 years (men<50 years: 9.77 ml × mmHg^−1^ × 100 (SD ± 2.96), men≥50 years: 6.23 ml × mmHg^−1^ × 100 (SD ± 3.21); women: <50 years: 7.86 ml × mmHg^−1^ × 100 (SD ± 2.76), women≥50 years: 4.53 ml × mmHg^−1^ × 100 (SD ± 2.58).

**Table 1:** Characteristics of participants at baseline.

	All *n* = 2362	Men *n* = 1186	Women *n* = 1176	*P*
Age, years (SD)	46 (11)	45.9 (11)	46.1 (11)	0.780
SBP, mmHg (SD)	119 (15)	122 (15)	116 (16)	<0.001
DBP, mmHg (SD)	70 (10)	71 (10)	68 (10)	<0.001
Pulse, bpm (SD)	63 (8)	63 (8)	64 (8)	<0.001
BMI kg/m² (SD)	26.4 (4.3)	26.7 (3.5)	26.2 (5)	0.004
Fasting glucose, mmol/l (SD)	5.3 (0.8)	5.4 (0.8)	5.2 (0.7)	<0.001
HOMA- IR median (IQR)	1.5 (0.8–1.8)	1.6 (0.85–1.94)	1.34 (0.75–1.63)	<0.001
Triglyceride, mmol/l median (IQR)	1.3 (0.8–1.5)	1.42 (0.87–1.72)	1.09 (0.7–1.29)	<0.001
LDL cholesterol, mmol/l (SD)	3.3 (0.9)	3.4 (0.9)	3.1 (0.9)	<0.001
CRP, mg/l median (IQR)	2.3 (0.7–2.4)	2.3 (0.7–2.2)	2.4 (0.7–2.6)	0.517
New-onset hypertension, n (%)	121 (5.1)	68 (5.7)	53 (4.5)	0.176
New-onset DM, n (%)	40 (1.7)	25 (2.1)	15 (1.3)	0.118
Current smoker, n (%)	444 (18.8)	186 (15.7)	258 (21.9)	<0.001
Non-drinker, n (%)	441 (18.7)	160 (13.4)	281 (23.9)	<0.001
Statin medication, n (%)	24 (1)	14 (12)	10 (9)	0.424
C2, ml × mmHg^−1^ × 100 (SD)	7.85 (3.39)	8.88 (3.42)	6.92 (3.09)	<0.001

*p* significance between men and women, *SBP* Systolic Blood Pressure, *DBP* Diastolic Blood Pressure, *BMI* Body Mass Index, *HOMA-IR* Homeostatic Model Assessment for Insulin Resistance, *LDL* Low-Density Lipoprotein, *DM* Diabetes Mellitus, *C2* Small Artery Elasticity, *IQR* interquartile range.

During the follow-up time, we observed 108 CV events. Of these, 70 events occurred in men (event rate: 5,9%), and 38 in women (event rate: 3.2%), with 11 of the 38 among those under 50 years. Most CV events occurred in individuals within the lowest quartile of C2 (Table 2).

**Table 2:** Events and event rates in each quartile of C2.

	Q1	Q2	Q3	Q4	All
All	591	591	590	590	2362
Fatal and non-fatal events	72	17	15	4	108
Event rate	12.2%	2.9%	2.5%	0.7%	5.4%
Incidence rate	823	194	172	46	309
Women	295	289	298	294	1176
Fatal and non-fatal events in women	24	6	6	2	38
Event rate for women	8%	2%	2%	0.7%	3.2%
Incidence rate in women	550	140	136	46	218
Men	296	302	292	294	1186
Fatal and non-fatal events in men	48	11	9	2	70
Event rate for men	16.2%	4.3%	3.4%	0.7%	7.1%
Incidence rate for men	1096	246	208	46	399

Q are quartiles based on levels of C2. Q4 highest quartile and Q1 is the lowest quartile.

The incidence rate is in cases/ 100000 person-year.

The number of all-cause mortality is 120 (event rate: 5.1%), and the CV mortality is 37 (event rate: 1.6%) under the same follow-up time.

**Fig. 2:**
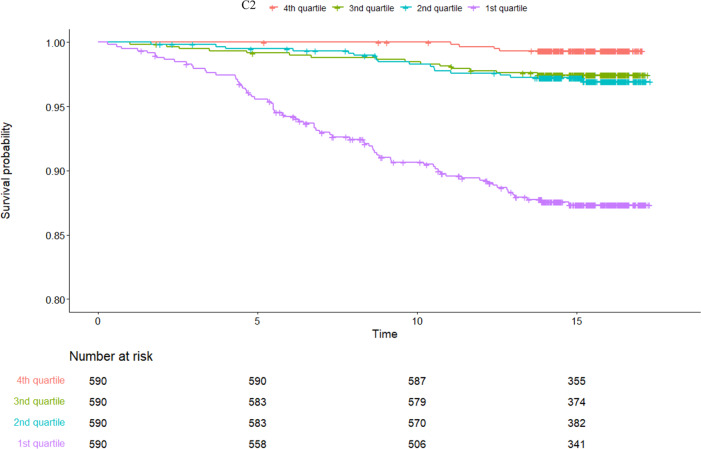
Kaplan-Meier analyses of the association between C2 quartiles (Q1–4) and incidence of CVD. (*N* = 2362, number of events=108, time in years).

Kaplan-Meier (KM) curves showed distinct differences between C2 quartiles regarding incident CVD (Fig. 2). In Cox- regression analysis, for each decrease in one quartile in C2, there was a significant increase in the risk for future CVD in the crude model (HR = 2.68, 95% CI: 2.12–3.39, *p* < 0.001) and the fully adjusted models (HR = 1.36, 95% CI: 1.01–1.82, *p* = 0.041) (Table 3). In a sensitivity analysis using pulse pressure instead of SBP, similar results were found in the fully adjusted model (HR = 1.47, 95% CI: 1.01–1.96, *p* = 0.01). When we added statin medication to the existing models, we got similar results to the main findings (HR = 1.35, 95% CI: 1.07–1.82, *p* = 0.045).

**Table 3:** Association between C2-quartiles and incidence of cardiovascular disease.

All (*N* = 2362, number of events = 108)			
	HR	95% CI for HR	p value
Crude data	2.68	2.12–3.39	<0.001*
Model 1	1.38	1.05–1.80	0.021*
Model 2	1.41	1.07–1.85	0.015*
Model 3	1.36	1.01–1.82	0.041*
**Men (*N*** = **1186, number of events** = **70)**			
	HR	95% CI for HR	p value
Crude data	2.94	2.17–3.99	<0.001*
Model 1	1.68	1.20–2.34	0.002*
Model 2	1.77	1.26–2.49	0.001*
Model 3	1.74	1.21–2.50	0.003*
**Women (*N*** = **1176, number of events** = **38)**			
	HR	95% CI for HR	p value
Crude data	2.34	1.62–3.37	<0.001*
Model 1	1.14	0.73–1.79	0.559
Model 2	1.14	0.72–1.78	0.581
Model 3	1.08	0.67–1.73	0.763
**Women over 50 years of age (*N*** = **338, number of events** = **27)**			
	HR	95% CI for HR	*p* value
Crude data	2.68	1.21–5.91	0.015*
Model 1	1.66	0.73–3.76	0.228
Model 2	1.64	0.72–3.71	0.239
Model 3	1.70	0.69–4.20	0.247

Model 1: Adjusted for age, sex, heart rate, and SBP.

Model 2: Adjusted as above and for HOMA- IR, LDL cholesterol, CRP.

Model 3: Adjusted as above and for smoking, alcohol consumption and physical activity.

*p* significance between C2 quartiles regarding CVD, *SBP* Systolic Blood Pressure, *HOMA-IR* Homeostatic Model Assessment for Insulin Resistance, *LDL* Low-Density Lipoprotein, *DM* Diabetes Mellitus, *CRP* C- reactive protein, *C2* Small Artery Elasticity.

In crude survival analyses, a significant association was abserved between quartiles of C2 and both CV mortality and all-cause mortality in the crude model (HR = 4.04, 95% CI: 2.41–6.80, *p* = <0.001 respectively HR = 2.86, 95% CI: 2.27–3.62, *p* = <0.001), i.e. lower C2 is associated with a higher risk of CV mortality and all-cause mortality. The significance was lost when we adjusted for age (HR = 1.36, 95% CI: 0.78–2.36, *p* = 0.276, respectively HR = 1.26, 95% CI: 0.97–1.63, *p* = 0.1).

In the sex-specific consistency analyses, a decrease in the C2-quartile was associated with an increased risk for CVD in men in the crude model (HR = 2.94, 95% CI: 2.17–3.99, *p* = <0.001) and after full adjustments (HR = 1.74, 95% CI: 1.21–2.50, *p* = 0.003). In women, similar results were observed in the crude model (HR = 2.34, 95% CI: 1.62–3.37, *p* < 0.001), but the associations were greatly attenuated after adjustments (HR = 1.08, 95% CI: 0.67–1.73 *p* = 0.763) (Table 3).

Most of the CVD events in women were registered over 50 years of age (under 50 years: *N* = 11, over 50 years: *N* = 27). In women above 50 years, quartiles of C2 were inversely associated with increased risk for future CVD in the crude model (HR = 2.68, 95% CI: 1.21–5.91, *p* = 0.015), but were attenuated after adjustments (HR = 1.7, 95% CI: 0.69–4.20) (Table 3).

When we use C2 quartiles as categorical variables in the model in the full sample, Cox regression shows a significant difference between C2 quartiles 1 and 4. There were significant differences in hazard ratio when we compared individuals at the lowest C2-quartile 1 with the other three quartiles (*p* < 0.001). These differences remained significant between C2 quartiles 1 and 4 after adjustments for possible confounding factors according to Model 3 (4^th^ quartile is the reference: 1st Quartile: HR = 4.27, 95% CI: 1.22–14.93, p = 0.023, 2nd Quartile: HR = 2.86, 95% CI: 0.82–10, 3rd Quartile: HR = 3.41, 95% CI: 0.97–12).

The C-statistics for model 3, including C2, was 0.844 with SE 0.019. After excluding C2, there was a minor decrease in C-statistics, i.e., 0.841 with SE 0.019.

## Discussion

In this study, we showed an independent association between C2 and cardiovascular morbidity and mortality regarding AMI and stroke in men. Similar results were found for postmenopausal women but were not statistically significant after adjustments.

Few studies have investigated the association between arterial elasticity and the development of CVD [3, 12, 24–28], and only a couple of studies are comparable with ours regarding population size [3, 12, 24, 25]. There are no studies with longer follow-up times and lower mean ages than ours. Similar to our results, lower small artery elasticity (equivalent of higher arterial stiffness) was associated with an increased risk for CVD in these studies.

In atherosclerosis, endothelial dysfunction occurs first [6], followed by structural changes [4] that appear parallel with atherosclerosis plaque formation long before myocardial ischemia and stroke develop. Coronary artery calcium, common carotid intima-media thickness, aortic distensibility and large and small arterial elasticity are structural and functional measures that characterize subclinical vascular diseases. These measures are independent predictors for incident arterial hypertension; C2 was the earliest predictor [29]. Moreover, apparent pathological changes in C2 [30] and pulse wave velocity [31] correlate with coronary artery disease severity, proven by coronary angiography. Our findings in this population-based study confirm that impaired small arterial elasticity increases the risk for CVD over time.

The major risk factors for reduced arterial elasticity, atherosclerosis, AMI and stroke are the same, not least because of mutual connections and dependencies. We adjusted for major cardiovascular risk factors such as age, sex, high blood pressure, impaired cholesterol levels, overweight and obesity, high blood glucose due to insulin resistance or DM, lack of physical activity, smoking and alcohol abuse.

The results for survival analyses regarding CV mortality and all-cause mortality are consistent with the main results, i.e., lower C2 is associated with a higher risk of CV morbidity and mortality. The young age at baseline and the limited number of CV-related deaths in these analyses makes it difficult to draw conclusions on the association between C2 and mortality.

In this study, we observed sex differences regarding arterial elasticity and in accordance with previous studies [32, 33], women had lower arterial elasticity than men [9, 12]. A component of these differences may be due to women’s smaller height [33], aortic size and diameter in vessels, although hormone differences may also explain these differences. Probably, more factors can contribute to sex differences regarding arterial elasticity, such as elastin-collagen alternation, vascular smooth muscle cell stiffening, oxidative stress, inflammation pathways and signaling in the renin-angiotensin-aldosterone system [32].

In women older than 50 years of age, we showed that small elasticity was lower than in women younger than 50 years of age. In this group, 27 cardiovascular events occurred, and we observed a similar association between C2, and the development of cardiovascular disease compared with men. In fact, effect sizes in these associations were very similar, even if the association became non-significant in women. It is in line with previous observations, arterial stiffness and CVD risk increase linearly in men, while women likely have a relative threshold in vascular aging, with a flatter line in the premenopausal stage and then a more rapid increase in arterial stiffening after menopause [32].

Analyses with C- statistics showed that C2 had a minor incremental role compared to common CV risk factors. The change in effect size was too small to conclude that the use of C2 would improve the predictive models in clinical practice. However, we note that lower values of C2 often precede increased blood pressure and so may be used as an early marker of increased risk. A low value of C2 should lead to increased surveillance and possibly to the use of blood pressure-lowering medication.

### Strengths and limitations

Both men and women participated in this study and a high participation rate was obtained. The same trained staff examined the participants according to a strict protocol. The data on CVD was obtained from reliable sources, namely the Swedish Cause of Death Register and the Swedish National Inpatient Register. Our study was observational. Thereby, it is not possible to conclude causality. The presence of data on many potential confounders permitted adjustments for several risk factors and lifestyle variables that influenced the association. However, residual confounding cannot be excluded. Most of the participants were Caucasians. The Swedish Cause of Death Register and the Hospital Discharge Register cover the whole study population and these registers are validated and have a high standard [34]. The study did not have specific data on menopausal transition and using 50/years of age as a cut-off was approximative. However, the mean age (SD) at natural menopause has been shown to be 50.8 (4.2) years in a large Swedish population study [35]. We note the limitations of this study in answering the question of the associations between C2 and CV mortality and all-cause mortality due to the limited number of events. A larger cohort in the older population might be more suitable to address that question.

### Clinical implications

We observed associations between C2 and CVD, where arterial elasticity predicts CVD independently of other known risk factors, including blood pressure in a general population without known diabetes mellitus or hypertension. It would be desirable to develop a simple measurement method for arterial elasticity that makes the technique easily accessible.

## Summary

### What is known about the topic

- Previous studies have shown that higher arterial stiffness predicts a higher risk for cardiovascular diseases.
- A few studies examine the predictive value of arterial elasticity for future cardiovascular diseases in the general multi-ethnic population.

### What this study adds

- Our study focuses on a younger population with a long follow-up time than previous studies.
- Showing that small arterial elasticity predicts cardiovascular disease independently of other known risk factors, including blood pressure in a general population in men, even at a young age and after adjustments for all possible confounders.

### Supplementary information

Supplementary table

## Data Availability

The datasets used and analysed during the current study are available from the corresponding author upon request.
