# Supplementary material for: Impaired artery elasticity predicts cardiovascular morbidity and mortality- A longitudinal study in the Vara-Skövde Cohort
Source: J Hum Hypertens. 2023 Oct 4;38(2):140–5. doi: 10.1038/s41371-023-00867-1 (PMC10844075; doi:10.1038/s41371-023-00867-1)
Supplement: Supplementary file 1 — Supplementary table [file 41371_2023_867_MOESM1_ESM.docx]

| Supplementary table: Characteristics for participants, divided by quartiles for C2 | | | | |
| --- | --- | --- | --- | --- |
|  | Q1 | Q2 | Q3 | Q4 |
| Number of participants | 591 | 591 | 590 | 590 |
| Age, years (SD) | 56.5 (11) | 45 (8.5) | 42.2 (7.3) | 40.2 (6.5) |
| SBP, mmHg (SD) | 132 (18) | 118 (12) | 114 (11) | 112 (10) |
| DBP, mmHg (SD) | 75 (11) | 70 (9) | 67 (9) | 66 (8) |
| Pulse, bpm (SD) | 65 (10) | 64 (9) | 63 (7) | 61 (6) |
| Fasting glucose, mmol/l (SD) | 5.5 (1.1) | 5.3 (0.6) | 5.2 (0.4) | 5.2 (0.5) |
| HOMA- IR median (IQR) | 1.6 (0.8-1.9) | 1.5 (0.8-1.8) | 1.4 (0.8-1.7) | 1.4 (0.8-1.6) |
| Triglyceride, mmol/l median (IQR) | 1.4 (0.9-1.7) | 1.3 (0.8-1.5) | 1.2 (0.7-1.4) | 1.2 (0.7-1.4) |
| LDL cholesterol, mmol/l (SD) | 3.6 (0.9) | 3.3 (0.9) | 3.1 (0.9) | 3.1 (0.9) |
| CRP, mg/l median (IQR) | 3 (0.9-3.1) | 2.1 (0.6-2.3) | 2.3 (0.6-2.2) | 1.9 (0.7-2) |
| New-onset hypertension, n (%) | 99 (16.8) | 16 (2.7) | 6 (1) | 0 |
| New-onset DM, n (%) | 23 (3.9) | 7 (1.2) | 4 (0.7) | 6 (1) |
| Current smoker, n (%) | 155 (26.2) | 108 (18.3) | 106 (17.9) | 75 (12.7) |
| Non-drinker, n (%) | 147 (24.8) | 102 (17.3) | 98 (16.6) | 94 (15.9) |
| Q are quartiles based on levels of C2. Q1 is the lowest quartile and Q4 is the highest quartile. | | | | |
|  | | | | |
